# Anti-neutrophil extracellular trap antibody in a patient with relapse of anti-neutrophil cytoplasmic antibody-associated vasculitis: a case report

**DOI:** 10.1186/s12882-018-0953-y

**Published:** 2018-06-22

**Authors:** Haruki Shida, Nobuhiro Hashimoto, Yoshihiro Kusunoki, Fumihiko Hattanda, Yayoi Ogawa, Terumasa Hayashi, Daigo Nakazawa, Sakiko Masuda, Utano Tomaru, Akihiro Ishizu

**Affiliations:** 1 Department of Rheumatology, Endocrinology and Nephrology, Faculty of Medicine and Graduate School of Medicine, Hokkaido University, Sapporo, Japan; 2 Departments of Kidney Disease and Hypertension, Osaka General Medical Center, Osaka, Japan; 3 Hokkaido Renal Pathology Center, Sapporo, Japan; 4 Faculty of Health Sciences, Hokkaido University, Kita-12, Nishi-5, Kita-ku, Sapporo, 0600812 Japan; 5 Department of Pathology, Faculty of Medicine and Graduate School of Medicine, Hokkaido University, Sapporo, Japan

**Keywords:** ANCA-associated vasculitis (AAV), Neutrophil extracellular traps (NETs), ANCA-NETs vicious cycle, Anti-NET antibody (ANETA)

## Abstract

**Background:**

Neutrophil extracellular traps (NETs) are web-like DNA decorated with antimicrobial proteins, such as myeloperoxidase (MPO), which are extruded from activated neutrophils. Although NETs are essential in innate immunity, an excessive formation of NETs has adverse effects, e.g., induction of anti-neutrophil cytoplasmic antibody (ANCA), to the hosts. Since ANCA can induce NET formation in the primed neutrophils, a positive feedback loop can be formed between NETs and ANCA, which is called “ANCA-NETs vicious cycle.”

**Case presentation:**

A 79-year-old Japanese woman developed hydralazine-induced pauci-immune necrotizing crescentic glomerulonephritis with MPO-ANCA. Although the illness improved after cessation of hydralazine, MPO-ANCA-associated vasculitis relapsed 16 months later. Remission was achieved 5 months after beginning of administration of prednisone. In order to determine the involvement of ANCA-NETs vicious cycle in this patient, we examined NET degradation and induction activities in sera obtained at the disease onset (Serum A; MPO-ANCA, 107 IU/ml), at relapse (Serum B; MPO-ANCA, 195 IU/ml), at 3 months after treatment (Serum C; MPO-ANCA, 4.5 IU/ml), and at remission (Serum D; MPO-ANCA, 2.4 IU/ml). NET degradation activity was low in the all sera. NET induction activity was high in Sera A, B, and C but not in D. Additionally, we demonstrated the presence of anti-NET antibody (ANETA) in Sera B and C but not in A or D.

**Conclusions:**

The collective findings suggest NET induction potential of ANETA in the present patient and that the ANETA could contribute to the enhancement of NETs resulting in amplification of the ANCA-NETs vicious cycle.

## Background

Neutrophil extracellular traps (NETs) are web-like DNA decorated with antimicrobial proteins, such as myeloperoxidase (MPO) and proteinase 3 (PR3), which are extruded from activated neutrophils [1]. NETs can trap microorganisms by the web-like DNA and kill them using the antimicrobial proteins. Chronic granulomatous disease (CGD) patients who cannot generate NETs are indeed susceptible to bacterial and fungal infections, and it was shown that restoration of NET formation in CGD resulted in resistance to such infections [2]. Although NET formation is regarded as an essential event in the innate immunity, an excessive formation of NETs can induce vascular endothelial cell damage and thrombosis resulting in the development of microvascular disorders [3, 4]. In addition, disordered regulation of NETs has been suggested to be involved in the pathogenesis of autoimmune diseases, including systemic lupus erythematosus (SLE) [5] and anti-thyroid drug propylthiouracil (PTU)-induced anti-neutrophil cytoplasmic antibody (ANCA)-associated vasculitis [6].

MPO-ANCA is the major pathogenic autoantibody in ANCA-associated vasculitis (AAV). Its pathogenicity has been demonstrated because the transfer of MPO-ANCA into wild-type mice resulted in the development of systemic small vessel vasculitis (SVV), including pauci-immune glomerulonephritis [7]. MPO-ANCA can activate neutrophils primed by pro-inflammatory cytokines, such as tumor necrosis factor-α (TNF-α), to release reactive oxygen species (ROS) and lytic enzymes and consequently injure small vessel endothelial cells [8, 9]. In addition, MPO-ANCA can induce NET formation in TNF-α-primed neutrophils [10].

One of the most important regulators of NETs is serum DNase I [5]. We have demonstrated that PTU-induced DNase I-resistant NETs can lead to the production of MPO-ANCA and subsequent development of SVV [6]. We have further demonstrated that the serum DNase I activity in patients with MPO-AAV is significantly lower than that in healthy controls [11]. Based on these findings, we have suggested that ANCA-NETs vicious cycle is involved in the pathogenesis of MPO-AAV.

We herein demonstrate a patient with hydralazine-induced MPO-AAV. The disease activity was controlled by immediate cessation of the causative drug, but MPO-AAV relapsed regardless of non-usage of the drug 16 months after the first diagnosis. To our knowledge, the relapse of drug-induced MPO-AAV is quite rare, especially in a situation without re-medication of the causative drug. We suspected that a certain immunological event occurred in the present patient. During relapse, anti-NET antibody (ANETA) was detected in the sera together with MPO-ANCA. We demonstrated that ANETA in the present patient could enhance NET formation and the possibility of its contribution to the amplification of the ANCA-NETs vicious cycle.

## Case presentation

A 79-year-old Japanese woman with a weight of 72 kg who has been maintained on anti-hypertensive drugs, including hydralazine, for more than 10 years, was advised on acute onset of proteinuria and microscopic hematuria by her family doctor. At this time, her serum creatinine (Cr) level was within normal range (0.8 mg/dl). One month later, however, the Cr level was elevated to 1.6 mg/dl. Therefore, she was referred to our hospital for admission.

**Fig. 1:**
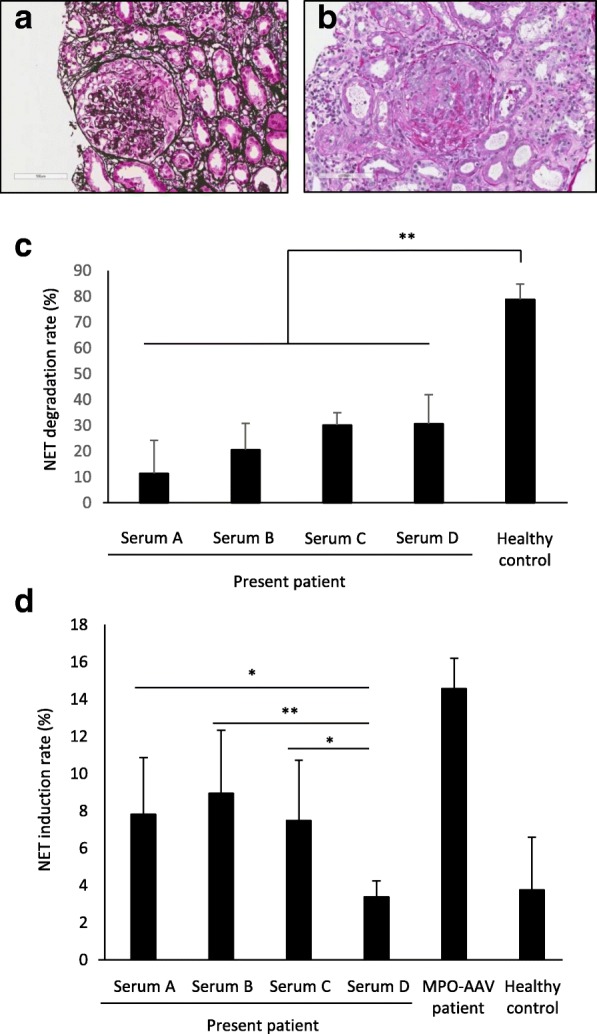
Renal biopsy findings and NET degradation and induction activities in sera. **a** Renal biopsy finding at the onset of disease. A total of 36 glomeruli, including 4 with global sclerosis, 17 with cellular crescent, 4 with segmental necrosis, and 11 intact glomeruli, were observed. A significant deposition of immunoglobulins (Ig) or complements was not evident (not shown). The representative glomerulus with cellular crescent formation is shown (Periodic acid methenamine silver with hematoxyline and eosin stain). Bar: 100 μm. **b** Renal biopsy finding at relapse of disease. A total of 18 glomeruli, including 6 with global sclerosis, 7 with cellular crescent, 1 with segmental necrosis, and 4 intact glomeruli, were observed. A significant deposition of Ig or complements was not evident (not shown). The representative glomerulus with cellular crescent formation is shown (Periodic acid Schiff stain, Bar: 100 μm). **c** NET degradation activity. Data are shown as the mean ± SD of sextuplicated results. **p < 0.01 in One-way ANOVA. Experiments were performed twice independently and we confirmed that the results were reproduced. **d** NET induction activity. Data are shown as the mean ± SD of sextuplicated results. *p < 0.05, **p < 0.01 in One-way ANOVA. Experiments were performed twice independently and we confirmed that the results were reproduced

On admission, vital signs revealed body temperature of 36.9 °C, blood pressure of 150/70 mmHg, and pulse rate of 80 per minute. The white blood cell count was 8700/μl with 2.0% eosinophils, red blood cell count was 307 × 10^4^/μl, and platelet count was 26.6 × 10^4^/μl. The following values indicated renal dysfunction; blood urea nitrogen: 25.0 mg/dl, Cr: 1.9 mg/dl, urinary protein: 2.5 g/day, and the presence of microscopic hematuria. Dysmorphic red blood cells were noted in the urine sample microscopically. In the serum, MPO-ANCA was 107 IU/ml (normal limit, 3.5 IU/ml), whereas C-reactive protein (CRP) was 0.2 mg/dl. PR3-ANCA and other ANCAs, including anti-elastase and anti-lactoferrin antibodies, were negative. The titer of anti-nuclear antibody (ANA) was less than 1:40. Anti-DNA antibody was negative. Complement values were as follows: C3 162.2 mg/dl (normal range, 71–135 mg/dl) and C4 37.7 mg/dl (normal range, 11–34 mg/dl). Renal biopsy revealed pauci-immune necrotizing crescentic glomerulonephritis (Fig. 1a).

Hydralazine-induced MPO-AAV was considered regardless of absence of skin involvement, elastase- and lactoferrin-ANCAs, anti-nuclear and anti-DNA antibodies, and hypocomplementemia, which are usually observed in the disease [12]. By discontinuation of the causative drug, the serum Cr level decreased gradually. At 10 months later, the serum Cr and MPO-ANCA levels recovered to 1.1 mg/dl and 13 IU/ml, respectively. Proteinuria and microscopic hematuria also disappeared. Since the clinical course was consistent with hydralazine-induced MPO-AAV and the serum CRP level was not high throughout the clinical course, no additional medication was administered.

After another 6 months of observation, the serum Cr and MPO-ANCA levels were re-elevated (Cr, 2.0 mg/dl; MPO-ANCA, 195 IU/ml) and proteinuria and microscopic hematuria were re-appeared. The titer of ANA was 1:40. Other ANCAs, including PR3-ANCA, and anti-DNA antibody were negative even at this time. Complement values were as follows: C3 149.4 mg/dl and C4 38.5 mg/dl. Renal biopsy was performed again and revealed cellular crescents in some glomeruli (Fig. 1b). These findings suggested the relapse of MPO-AAV. Since she had fever (38.3 °C), and the serum CRP level was elevated to 10.0 mg/dl at this time, administration of 30 mg prednisone (0.5 mg/kg body weight) was initiated. The illness improved rapidly, and remission was achieved 5 months after the beginning of treatment. The patient has remained in remission thereafter (Table 1).

**Table 1:** Medical History Timeline

AAV clinical course	Months after diagnosis	MPO-ANCA (IU/ml)	Serum
Disease on set, Diagnosis	0	107	A
Cessation of hydralazine	10	13	–
Cessation of hydralazine, Relapse	16	195	B
Administration of prednisone	19	4.5	C
Administration of prednisone, Remission	21	2.4	D

### Serological analyses

This study was approved by the Ethical Committee of Osaka General Medical Center (Permission No. 29-C0313) and the Ethical Committee of Faculty of Health Sciences, Hokkaido University (Permission No. 15–90). After acquisition of written informed consent from the patient, serum samples were obtained at the disease onset (Serum A; MPO-ANCA, 107 IU/ml), at relapse (Serum B; MPO-ANCA, 195 IU/ml), at 3 months after treatment (Serum C; MPO-ANCA, 4.5 IU/ml), and at remission (Serum D; MPO-ANCA, 2.4 IU/ml).

To assess the involvement of NETs in the pathophysiology of this patient, we determined the NET degradation activity in the serum samples at first. In brief, peripheral blood neutrophils from a healthy volunteer were seeded in slide chambers (1 × 10^6^/ml), incubated for 15 min at 37 °C, and then made to react with 100 nM phorbol myristate acetate (PMA; Sigma-Aldrich, St. Louis, MO) for 3 h at 37 °C. We have confirmed that this stimulation induces NETs conspicuously [11]. After washing with PBS, the cells were incubated in 10% Serum A, B, C, or D for 6 h at 37 °C. For positive control, 10% serum of a healthy volunteer (49 years old, male) was employed. This sample exhibited the average value for NET degradation in our previous study [11]. To stop the serum nuclease activity, 2 mM EDTA was added, and then the remaining cells on the slides were fixed with 4% paraformaldehyde (PFA) followed by mounting with the solution containing DAPI. Photomicrographs (magnification, × 200) were taken randomly under a fluorescent microscope (6 fields/well of chamber slides), and then the residual NET area was determined using Image J software. NET degradation rate (%) was calculated as follows; {(residual NET area, incubated with PBS) – (residual NET area, incubated with serum) / (residual NET area, incubated with PBS)} × 100. As a result, the NET degradation activity was entirely low in Sera A, B, C, and D compared with the healthy control (Fig. 1c). Correspondingly, the DNase I activity as determined using ELISA kit (Orgentec GmbH, Mainz, Germany) was low in Sera A (21.7%), B (28.3%), C (22.8%), and D (33.5%) compared with the healthy controls {mean ± standard deviation (SD), 52.6 ± 12.1%}.

Next, we determined the NET induction activity of IgG, which was isolated from the serum samples, using immunoadsorbent columns (Protein G HP SpinTrap, GE Healthcare, Tokyo, Japan). Contamination of endotoxin in the IgG samples was ruled out using the Limulus test kit (Wako Pure Chemical, Osaka, Japan). Peripheral blood neutrophils from a healthy volunteer were seeded in slide chambers (1 × 10^6^/ml), pre-treated with 5 ng/ml TNF-α for 15 min at 37 °C to express MPOs on the cell surface, and then made to react with 250 μg/ml of the IgG samples. Serum IgG samples from a 65-year-old woman patient with MPO-AAV (MPO-ANCA, 93.2 IU/ml) and the healthy volunteer were employed as positive and negative controls, respectively. These samples exhibited the average values for NET induction in our previous study [11]. After incubation for 3 h at 37 °C, the supernatants were removed and the remaining cells on the slides were fixed with 4% PFA. Finally, the remaining cells were mounted with the DAPI-containing solution. Photomicrographs (magnification, × 200) were taken randomly under a fluorescent microscope (6 fields/well of chamber slides), and then the rates of NET-forming neutrophils were determined using ImageJ software. As a result, the NET induction activity was high in Sera A, B, and C, whereas that in Serum D was equivalent to the healthy control (Fig. 1d).

**Fig. 2:**
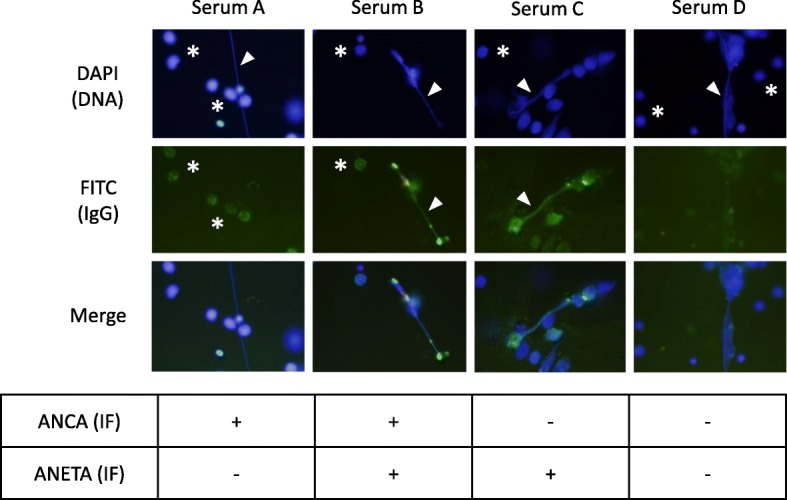
Detection of ANETA. Under the experimental condition, approximately 40% of neutrophils formed NETs (arrowheads in DAPI panels), whereas the others sustained the cell morphology (asterisks in DAPI panels). ANCA was detected in Sera A and B (asterisks in FITC panels) but not in Serum C or D. ANETA was detected in Sera B and C (arrowheads in FITC panels) but not in Serum A or D. Representative photomicrographs are shown

Lastly, we conducted immunofluorescent (IF) tests to determine the presence of ANETA in the serum samples. Briefly, peripheral blood neutrophils from a healthy volunteer were seeded in slide chambers (1 × 10^6^/ml), incubated for 15 min at 37 °C, and then made to react with 20 nM PMA for 2 h at 37 °C. After washing with PBS, the cells were fixed with 4% PFA, and then made to react with 250 μg/ml of the IgG samples for 1 h at 37 °C. After washing with PBS, the cells were next allowed to react with 1:5000 dilution of FITC-conjugated anti-human IgG antibodies for 1 h at 37 °C followed by mounting with the solution containing DAPI. As shown in Fig. 2, ANCA was detected in Sera A and B but not in Sera C or D; thus, these findings were consistent with the ELISA titers of MPO-ANCA. On the other hand, ANETA was detected in Sera B and C but not in Serum A or D.

## Discussion

We are demonstrating a patient who developed hydralazine-induced MPO-AAV. At the onset of the disease, necrotizing crescentic glomerulonephritis occurred with a high titer of MPO-ANCA (107 IU/ml). By cessation of hydralazine, the serum Cr level and MPO-ANCA titer decreased gradually. These findings suggest the pathogenicity of MPO-ANCA induced by hydralazine in the present patient.

Although the patient recovered from the illness by discontinuation of hydralazine, MPO-ANCA remained at the titer of 13 IU/ml 10 months later. Afterwards, MPO-AAV relapsed regardless of non-usage of hydralazine 16 months after the first diagnosis. It is speculated that the persistent NETs could lead to the re-elevation of MPO-ANCA in the present patient according to the following observations. 1) The baseline potential of NET degradation also represented by DNase I activity in the serum was low. 2) MPO-ANCA has been shown to induce NETs [10]. 3) The excessive formation of NETs has been shown to lead to MPO-ANCA production [6, 13]. These are consistent with the concept of the ANCA-NETs vicious cycle in MPO-AAV [11].

In addition to MPO-ANCA, ANETA was detected in the serum at relapse of MPO-AAV. Interestingly, NET induction potential of IgG samples derived from the serum at 3 months after treatment (Serum C, ANCA^−^/ANETA^+^ in IF test) was high. Therefore, these findings suggest NET induction potential of ANETA in the present patient. ANETA possibly cooperates with MPO-ANCA to amplify the ANCA-NETs vicious cycle and contributes to relapse of the disease. Although this hypothesis should be confirmed by future studies, the concept is consistent with the recent report of in vivo demonstration of NETs in patients with hydralazine-induced AAV [14].

Currently, antigens of ANETA remain unrevealed. MPO is a component of NETs; however, MPO-ANCA in Serum A did not bind to NETs in the IF test. This finding suggests the masking of the MPO epitope in the NETs, which is recognized by MPO-ANCA in Serum A. On the contrary, IgG in Serum C was bound to NETs but not to neutrophils that conserved their morphology. The collective findings suggest the difference of ANETA from MPO-ANCA, though the possibility that ANETA recognizes the modified MPO cannot be ruled out for the present. In addition, Roth et al. have demonstrated that the anti-MPO peptide (amino ascids 447–459) antibody is masked by the ceruloplasmin derivative in serum samples [15]. Therefore, the possible identity of the ANETA and anti-MPO peptide (amino ascids 447–459) antibody cannot be ruled out, either.

Hakkim et al. demonstrated that anti-DNA antibodies were detected as ANETA in some SLE patients and suggested that these antibodies could interfere with the DNase I action resulting in the impaired regulation of NETs [5]. Since anti-nuclear and anti-DNA antibodies were not detected during the clinical course, ANETA in the present patient was different from autoantibodies against nuclear components.

The best used reagent to induce NETs is PMA, which activates the Raf-MEK-ERK cascade, NADPH oxidase-dependent production of ROS, and receptor-interacting protein kinase/mixed lineage kinase domain-like-mediated signals [16, 17]. In this pathway, peptidylarginine deiminase 4-dependent citrullination of histones induces decondensation of DNA resulting in a mixture of DNA and antimicrobial proteins, which are contained originally in intracytoplasmic granules [18]. Thereafter, these substances are extruded from the ruptured plasma membrane. It is conceivable that molecules are modified in NET-forming neutrophils resulting in alteration of their antigenicity from the unstimulated condition. Accordingly, we have to consider the possibility that epitopes of degraded and/or complexed molecules are recognized by ANETA.

Furthermore, ANETA probably includes various antibodies that demonstrate diverse specificity and functions, e.g., inhibitory action against DNase I and NET induction property. Further studies are needed to determine the specificity and significance of ANETA.

## Conclusion

Through the experience gained from the management of this patient, we have learned that ANETA is worthy of attention for understanding the pathophysiology of neutrophil-related autoimmune diseases, especially AAV.

## Abbreviations

AAV: ANCA-associated vasculitis
ANA: Anti-nuclear antibody
ANCA: Anti-neutrophil cytoplasmic antibody;
ANETA: Anti-NET antibody
CGD: Chronic granulomatous disease
Cr: Creatinine
CRP: C-reactive protein
IF: Immunofluorescent
Ig: Immunoglobulins
MPO: Myeloperoxidase
NETs: Neutrophil extracellular traps
PFA: Paraformaldehyde
PR3: Proteinase 3
PTU: Propylthiouracil
ROS: Reactive oxygen species
SLE: Systemic lupus erythematosus
SVV: Small vessel vasculitis
TNF: Tumor necrosis factor
